# UHPLC-ESI-Q*q*TOF Analysis and In Vitro Rumen Fermentation for Exploiting *Fagus sylvatica* Leaf in Ruminant Diet

**DOI:** 10.3390/molecules27072217

**Published:** 2022-03-29

**Authors:** Marialuisa Formato, Simona Piccolella, Christian Zidorn, Alessandro Vastolo, Serena Calabrò, Monica Isabella Cutrignelli, Severina Pacifico

**Affiliations:** 1 Department of Environmental, Biological and Pharmaceutical Sciences and Technologies, University of Campania ‘Luigi Vanvitelli’, Via Vivaldi 43, 81100 Caserta, Italy; marialuisa.formato@unicampania.it (M.F.); simona.piccolella@unicampania.it (S.P.); 2 Pharmazeutisches Institut, Abteilung Pharmazeutische Biologie, Christian-Albrechts-Universität zu Kiel, Gutenbergstraße 76, 24118 Kiel, Germany; czidorn@pharmazie.uni-kiel.de; 3 Department of Veterinary Medicine and Animal Production, University of Naples Federico II, Via Federico Delpino, 1, 80137 Napoli, Italy; alessandro.vastolo@unina.it (A.V.); serena.calabro@unina.it (S.C.); monica.cutrignelli@unina.it (M.I.C.)

**Keywords:** *Fagus sylvatica* L., UHPLC-ESI-Q*q*TOF analysis, polyphenols, flavonoids, in vitro rumen fermentation, volatile fatty acids

## Abstract

In recent years, animal husbandry has aimed at improving the conditions of livestock animals useful for humans to solve environmental and health problems. The formulation of animal feeds or supplements based on antioxidant plant compounds is considered a valuable approach and an alternative for livestock productivity. Forest biomass materials are an underestimated source of polyphenolic compounds whose sustainable recovery could provide direct benefits to animals and, indirectly, human nutrition. In this context, an alcohol extract from leaves of *Fagus sylvatica* L. was first investigated through an untargeted ultra-high-performance liquid chromatography–high-resolution tandem mass spectrometry (UHPLC-HRMS/MS) approach. Then, it was fractionated into a fatty acid-rich and a polyphenolic fraction, as evidenced by total lipid, phenol, and flavonoid content assays, with antiradical and reducing activity positively correlated to the latter. When tested in vitro with rumen liquor to evaluate changes in the fermentative parameters, a significant detrimental effect was exerted by the lipid-rich fraction, whereas the flavonoid-rich one positively modulated the production of volatile fatty acids (i.e., acetate, butyrate, propionate, etc.).

## 1. Introduction

The growing prevalence of chronic diseases and the adoption of animal husbandry for greater revenue and productivity, together with the new emphasis on livestock health, are driving the development of innovative ruminant feeds able to improve animal productivity and to feasibly reduce the footprint of the entire food/feed system on the environment and climate [1,2,3]. The European project “Rumen-Up”, through the creation of sustainable plant-based solutions, highlighted, almost in a pioneering way, that plants or their parts could be used for livestock feed and to manipulate rumen fermentation, providing benefits to humans, as the modulation of rumen microbial fermentation markedly affects the lipid composition of milk and meat [4]. Thus, the recovery of new feed ingredients from agro-industrial byproducts and residual forest tree materials [5,6] is currently being explored as a valuable strategy to achieve sustainable animal production through the efficient use of resources and waste reduction [7,8], which is also requested by Agenda 2030 (goal 12; that is, “Responsible Consumption and Production: Reversing current consumption trends and promoting a more sustainable future”).

As recently reported [9], grape pomace, olive byproducts and citrus pulp are just a few examples of sources of high added value ingredients for sustainable livestock diets [10,11,12]. In the research and development of products that can contribute to reducing the environmental impact and safeguarding the environment, the valorization of chestnut and quebracho biomasses is actually applied for livestock, and several plant extracts diversely composed in specialized metabolites, such as tannins, saponins, and flavonoids, as well as in essential oils [13,14], are of interest. Hydrolysable and condensed tannins are especially considered as antimicrobial feed additives, due to their antibacterial and antiparasitic activity [15], whereas saponins are reported to affect rumen defaunation and to impair protein digestion [16]. In general, these non-nutritive compounds are able to modify ruminal fermentation characteristics, to inhibit ruminal methanogenesis, and to enhance livestock performance, overall representing an alternative to antibiotic feed additives [17,18,19]. In addition, plant material to be disposed that is rich in flavonoids might be an interesting source for novel food additives. Flavonoids are broadly known as antioxidant and anti-inflammatory compounds. Therefore, and due to their related precious benefits under a variety of stressful conditions, these compounds have received a lot of attention for productive performance and health [20]. Moreover, flavonoids might beneficially interact with rumen microbiota and impact carbohydrate fermentation, protein degradation, and lipid metabolism.

Different effects on the microbiome of ruminant digestive systems, whose microorganisms are responsible for feed transformation into products, are reported. Complex carbohydrates are broken down into simple sugars by means of microbial fermentation, and sugar monomers could be used as an energy source and for biosynthesising products, such as volatile fatty acids (VFAs), methane, and carbon dioxide [21]. In fact, due to their antioxidant efficacy, polyphenols could prevent lipoperoxidation and/or protein degradation [22,23]. Furthermore, research is pushing forward to manipulate farm animal feed to influence the sensory, nutritional, and technological characteristics, increasing the level of bioactive metabolites [24]. Additionally, plant extracts open up a fascinating scenario for enhancing both livestock productivity and dairy products (e.g., milk, meat, and cheese) quality. Indeed, different factors, mainly including the plant source and its chemical composition, which can vary based on harvesting time and/or extraction method applied, could affect the efficacy of plant extracts, impoverishing the in vivo applicability. Thus, in order to fully exploit plant compound diversity, a deepening insight into the chemical compositions of plant extracts and the optimization of fractionation procedures could be pursued to differently concentrate phytochemicals on the basis of their features (i.e., polarity, solubility, etc.). This is in line with a performing use of the different coexisting specialized metabolites for feed/food purposes.

In this context, based on a previous phytochemical investigation on *F. sylvatica* leaves [25] as a renewable source for feed and food, herein, a two-steps fractionation process has been proposed, achieving two main fractions. The latter, together with their parental extract, were preliminarily screened for their total phenolic (TPC), flavonoidic (TFC), and lipidic contents (TLC), as well as for their antiradical and reducing activity. The further chemical investigation by means of UV–Vis spectroscopy and ultra-high-performance liquid chromatography coupled to quadrupole time-of-flight tandem mass spectrometry (UHPLC-Q*q*TOF-MS/MS) analyses opened up the evaluation of their effect on in vitro ruminal fermentation (cumulative gas production; organic matter degradability; fermentation kinetics; and end products, i.e., ammonia-N, volatile fatty acids, branched-chain fatty acid proportion, and acetate/propionate ratio). To the best of our knowledge, such a rigorous approach, in which the bioactivity assessment is closely coupled to a detailed chemical investigation, has never been reported in the literature before.

## 2. Results and Discussion

**Figure 1:**
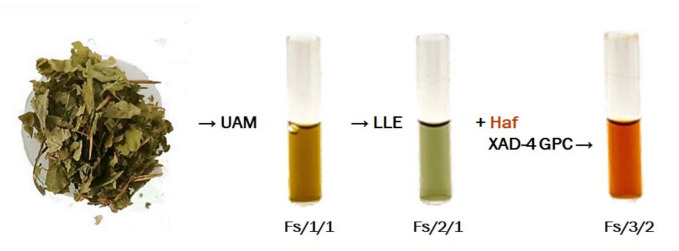
Extraction and fractionation of *F. sylvatica* L. leaf. UAM: ultrasound assisted maceration; LLE: liquid–liquid extraction; Haf: hydroalcoholic fraction; XAD-4 GPC: Gel Permeation Chromatography on Amberlite XAD-4 absorbent resin.

The alcoholic extract of *F. sylvatica* leaf (Fs/1/1), whose chemical profiling was previously reported [25], consisted of an abundant polyphenol part and fatty acids. Polyphenols were mainly flavonols, beyond hydroxycinnamoyl compounds, while mono- or polyhydroxylated fatty acids were identified, also according to the literature [26], at the highest retention time in the total ion chromatogram [25]. The Fs/1/1 extract exhibited a good antioxidant efficacy, which was supposed to be enhanced following fractionation and with the obtainment of fractions differently composed in terms of polarity and identity of their specialized metabolites. In particular, with the aim to achieve a fraction depauperated in fatty acids, a biphasic extraction was firstly employed on Fs/1/1, and the nonpolar fraction Fs/2/1 was partitioned from an hydroalcoholic fraction comprising hydrophilic metabolites. This latter was further chromatographed using XAD-4 polystyrene resin, suitable for the recovery of polyphenols (Figure 1). Thus, two organic fractions were collected (Fs/2/1 and Fs/3/2), and with the aim to explore the impact on microbial fermentation processes of the parental leaf extract and its most bioactive fractions, a spectrophotometric screening was first carried out. 

**Figure 2:**
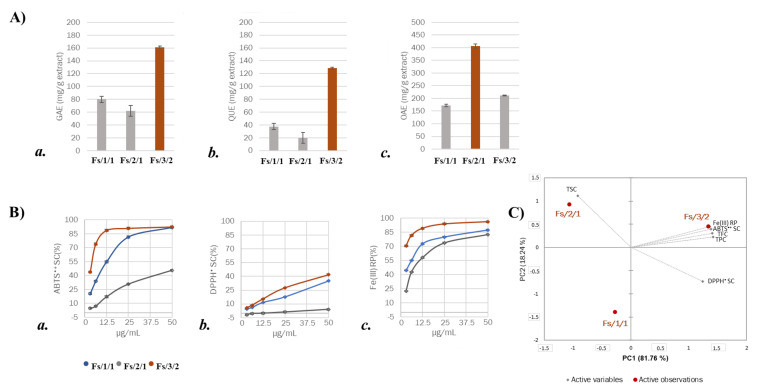
(**A**) (**a**) Total phenolic content (TPC), expressed as mg of gallic acid equivalents (GAE) per g of extract; (**b**) total flavonoidic content (TFC), expressed as mg of quercetin equivalents (QUE) per g of extract; (**c**) total lipidic content (TLC), expressed as mg of oleanolic acid equivalents per g of extract. Values reported are the mean ± SD of three independent measurements. (**B**) (**a**) Scavenging capability (SC%) vs. 2,2′-azino-bis(3-ethylbenzothiazoline)-6-sulfonic acid (ABTS) radical cation, (**b**) scavenging capability (SC%) vs. 2,2-diphenyl-1-picrylhydrazy (DPPH) radical, and (**c**) Fe (III) reducing power (RP) of *F. sylvatica* extract and organic fractions therefrom. Values reported are the mean ± SD of three independent measurements. (**C**) PCA analysis based on colorimetric compositive assays and data from antiradical and reducing power tests.

The preliminary assessment of the total phenolic (TPC), total flavonoidic (TFC), and total lipidic contents (TLC) markedly differentiated the fractions Fs/2/1 and Fs/3/2. This latter appeared mainly constituted by phenol compounds and flavonoids, with the highest TPC and TFC values equal to 161.3 ± 15.0 gallic acid equivalents (GAEs) and 128.4 ± 1.9 mg quercetin equivalents (QUEs) per g of extract, respectively (Figure 2A(a,b)). The lipidic content of Fs/3/2 was not negligible, so much so that it was estimated to be equal to 212.0 ± 27.5 mg oleanolic acid equivalents (OAEs) per g of extract. It could be due to the saponins previously identified. In fact, the assay employed originally utilized to colorimetrically evaluate saponins was enlarged in its aims, as the presence of double bonds or free hydroxyl groups within lipid analytes is a feature that allows the positive response to be detected [27,28].

The organic fraction Fs/2/1 likely lacked phenolic compounds (62.2 ± 8.6 mg QUEs per g of extract) and, specifically, flavonoid constituents (19.7 ± 3.2 mg QUEs per g of extract), being richer in lipid compounds (Figure 2A). To further corroborate the diverse chemical constitution and to preliminarily discriminate the fractions based on their antiradical and reducing activities, DPPH (2,2-diphenyl-1-picrylhydrazyl) and ABTS (2,2′-azinobis-(3-ethylbenzothiazolin-6-sulfonic acid) assays, as well as the Fe(III) reducing test, were performed. In this context, the data acquired highlighted that fraction Fs/3/2 was effective in scavenging both ABTS^●+^ and DPPH^●^ with ID_50_ values equal to 0.74 ± 0.08 µg/mL and 40.8 ± 1.1 µg/mL, respectively. Furthermore, it was able to reduce ferric ions also at the lowest tested dose, exhibiting a Trolox Equivalent Antioxidant Capacity (TEAC) value equal to 28.3. All data underwent a Principal Component Analysis, and the main components obtained (PC1 and PC2) allowed us to further interpret the original dataset. In particular, it was observed that phenol and flavonoid contents, as well as antiradical and reducing activity, were positively correlated to the fraction Fs/3/2, whereas the lipid content represented the main feature of the fraction Fs/2/1. 

### 2.1. Chemical Investigation on Fs/2/1 and Fs/3/2 Fractions

The fractions Fs/2/1 and Fs/3/2 were chemically investigated by means of UV spectroscopy and UHPLC-ESI-QqTOF tandem mass spectrometry.

UV data, with absorption bands at 235 and 205 nm, highlighted that the main constituents of the Fs/2/1 fraction could be fatty acids and their oxidized derivatives. Indeed, the fraction also showed very weak UV absorption at 328, 415, and 667 nm, in line with the trace presence of phenol, carotenoid, and chlorophyll compounds. The UV spectrum of the Fs/3/2 fraction, with bands at 330, 295, 270, and 203 nm, was in line with the occurrence of hydroxycinnamoyl compounds, flavonoids, and saponins (Figure S1).

**Figure 3:**
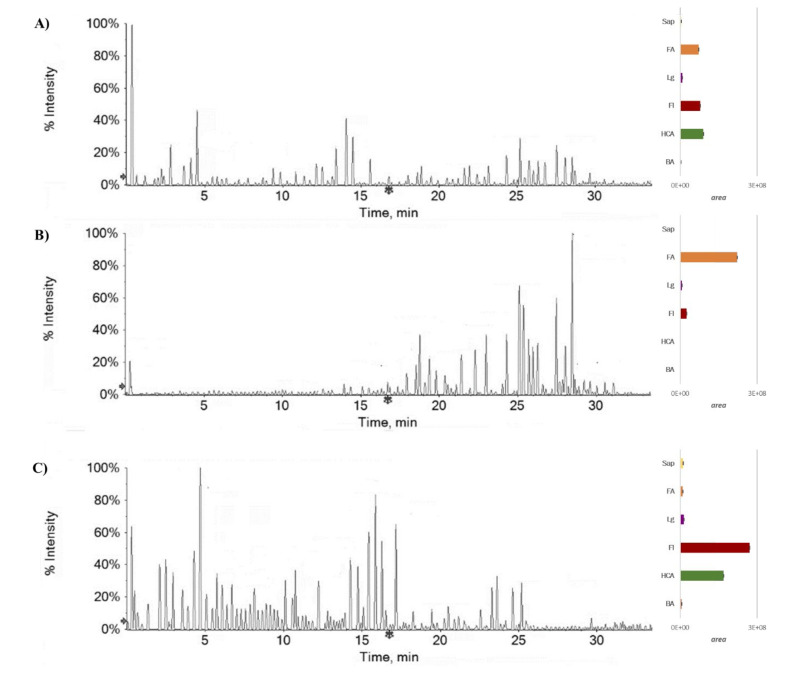
Total ion current (TIC) chromatograms of Fs/1/1 extract (**A**), Fs/2/1 fraction (**B**), and Fs/3/2 fraction (**C**). The relative content of each extract/fraction in a derivative of benzoic acid (BA) and hydroxycinnamic acid (HCA), as well as of flavonoids (Fl), lignans (Lg), fatty acids (FA), and saponins (Sap), is shown next to each chromatogram.

UHPLC-QqTOF-MS/MS analysis, and the relative quantifications relying on the change of the levels of each identified compound (Table 1) in the parental Fs/1/1 extract and its fractions Fs/2/1 and Fs/3/2 (Figure 3) were carried out. It appeared that, according to fractionation-induced reduction of the plant extract complexity, Fs/2/1 mainly constituted octanoids and other fatty acids derivatives, where Fs/3/2 massively contained the phenol and polyphenol components of the parental extract, with a few differences. Thus, the polar constituents of beech methanolic extract were part of Fs/3/2, in which minor constituents could be also masked, such as a galloyl hexoside with the [M-H]^−^ at *m*/*z* 331.0667 and a hydroxybenzoyl hexoside whose deprotonated molecular ion was at *m*/*z* 299.0770. 

Dihydroxybenzoic acid hexoside ([M-H]^−^ at *m*/*z* 315.722) and a hydroxyphenylacetic acid hexoside ([M-H]^−^ at *m*/*z* 313.0939) was also recognized. However, hydroxycinnamoyl derivatives and flavonoids were the main compounds of the Fs/3/2 fraction. Among hydroxycinnamoyl-based compounds, 3-O- and 5-O-caffeoyl quinic acids (**7**, **8**, **19**, and **22**) were identified, together with 3-O- and 5-O-p-coumaroyl quinic acids (**12**, **13**, **25**, and **28**). Two isomers of caffeoyl shikimic acids were first-time distinguishable as 4-O-CSA (**27**), based on the base peak at *m*/*z* 161.024, and 5-O-CSA (**29**), whose TOF-MS/MS base peak was at *m*/*z* 179.0350, respectively [29], and different caffeoyl and p-coumaroyl esters of threonic acid (**9**, **11**, **16**, **18,** and **20**). Threonic acid, which is a product of ascorbate catabolism [30], was identifiable thanks to the neutral loss of 118.02 and the fragment ion at *m*/*z* 117.02. The hydroxycinnamoyl moiety of the threonic acid depsides was identified based on the characteristic deprotonated ion, which was detected at *m*/*z* 179.03 for caffeoate and at *m*/*z* 163.04 for p-coumarate, and also based on their relative decarboxylated ions, which were, respectively, at *m*/*z* 135.03 [caffeic acid–CO_2_–H^+^]^−^ and 119.05 [p-coumaric acid–CO_2_–H^+^]^−^.

Caffeoyl esters of threonic acid, in which the hydroxycinnamoyl moiety was at carbons C2 or C4 of the threonic acid, were found in Chelidonium majus L., an herbal drug traditionally used against diseases of the liver, the gallbladder, and various skin disorders [31], and 4-E-caffeoyl-L-threonic acid was observed as an oviposition stimulant for Papilio bianor from Orixa japonica leaves [32]. Coumaroyl malic acids (**26** and **30**) were also herein tentatively identified, with the malate ion appearing at *m*/*z* 133.0301 on the TOF-MS/MS spectrum. Other hydroxycinnamoyl compounds were the hexoside of caffeic acid (**10**, with the deprotonated molecular ion at *m*/*z* 341.0874), and coumaric acid (**15**, [M-H]^−^ at *m*/*z* 325.0926) and, again, caffeoyl propionic acid (**21**) and caffeic acid (**17**).

Flavonoids accounted for a large part of Fs/3/2. Among them, a procyanidin B-type dimer (**14**), whose deprotonated molecular ion at *m*/*z* 577.1353 provided fragment ions at *m*/*z* 451.1046, 425.0883, 407.0781, and 289.0711; a flavanone O-glycoside (**23**); and three flavanone C-glycosides (**31**, **32**, and **38**) and flavonol glycosides were the main constituents. Myricetin (in glycoside **33**), quercetin, and kaempferol were found to be the core aglycones, with kaempferol glycosides as the most abundant compounds.

Quercetin 3-O-galactopyranoside (**34**), quercetin 3-O-glucopyranoside (**36**), quercetin glucuronide (**35**), a quercetin 3-O-pentoside (**41**), and quercetin 3-O-acetylhexose (**43**) were constituents of the fraction. Neutral losses of 162.05 Da (dehydrated hexose) and 132.04 Da (dehydrated pentose), 146.06 (dehydrated deoxyhexose), 204.07 Da (dehydrated acetylhexose), and 176.03 (dehydrated hexuronic acid) suggested the glyconic moiety identity.

Kaempferol 3-O-glucopyranoside (**42**), 3-O-galactopyranoside (**45**), and two kaempferol 3-O-pentosides (**46**, **47**) were identifiable based on a relative neutral loss of 162.05 Da (hexose) and 132.04 Da (pentose) from the deprotonated molecular ion and the formation of the abundant [aglycone-H]^●−^ ion at *m*/*z* 284.03 (base peak), whose intensity was diagnostic for the localizing glyconic moiety.

A kaempferol deoxyhexoside (**49**) was also tentatively identified, whereas the detected [M-H]^−^ ion at *m*/*z* 489.1049 for compound **48** and its relative TOF-MS/MS spectrum were in accordance with the acetyl derivative of kaempferol hexoside. Coumaroyl kaempferol glycosides (**51**, **64**, **65**, **66**, **73**, and **74**) were also poorly detected, together with the kaempferol hexuronides (**37** and **44**) and the kaempferol aglycone (**52**). Compounds belonging to the neolignan class (**39**, **40**, **50**, and **55**) were detected, although the TOF-MS/MS low fragmentation did not allow to unambiguously identify them. Oleanolic acid-based saponins **68**, **69**, **71**, and **72** appeared to be fully transferred into this fraction.

**Figure 4:**
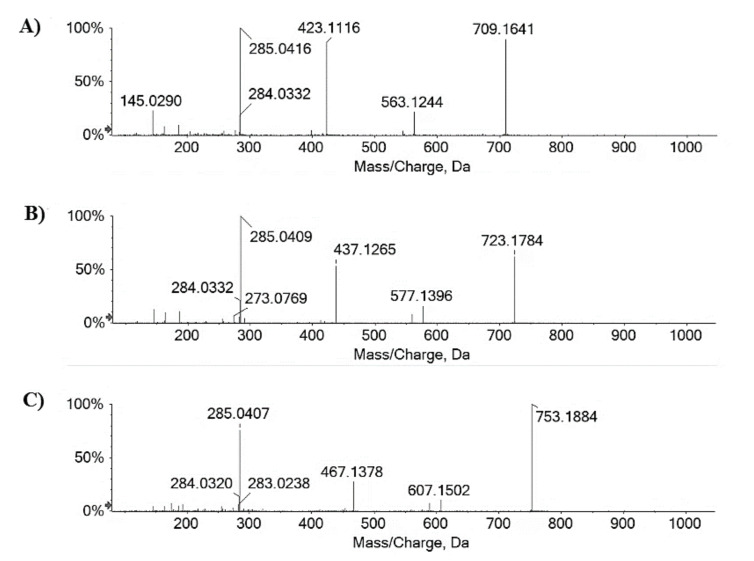
TOF-MS/MS spectra of the acylated kaempferol glycosides (**A**) **66**, (**B**) **73**, and (**C**) **74** with [M-H]^−^ ions at *m*/*z* 709.1463, 723.1720, and 753.1825, which were detected in the lipophilic Fs/2/1 fraction.

Kaempferol glycosides and acylated derivatives were found also in the apolar Fs/2/1 fraction, which mainly contained octadecanoids and other fatty acid derivatives. In particular, traces of kaempferol hexoside, pentoside, and deoxyhexoside were tentatively identified, whereas kaempferol p-coumaroyldeoxyhexosides (at *m*/*z* 577.1368 and 577.1357; **64** and **65**, respectively), di-p-coumaroyldeoxyhexoside (at *m*/*z* 723.1720; **73**), and p-coumaroyl feruloyl deoxyhexoside (at *m*/*z* 753.1825; **74**), as well as kaempferol di-p-coumaroylpentoside (at *m*/*z* 709.1463, **66**), eluting at higher retention times, were the most abundant. In Figure 4, TOF-MS/MS spectra of compounds **66**, **73** and **74** are reported. Neutral losses of 146.04 Da (dehydrated p-coumaric acid), 292.09 Da (p-coumaroyldeoxyhexose-H_2_O), and 438.13 Da (di-p-coumaroyldeoxyhexoside-H_2_O) suggested the tentative identification of kaempferol p-coumaroyl- and di-p-coumaroyl deoxyhexoside.

The interest in these compounds is mainly related to their antimicrobial activity, so much so that antibacterial kaempferol coumaroyl rhamnosides, commonly called platanosides, were previously isolated from the leaves of Platanus occidentalis [33]. Kaempferol-3-O-(bis-p-coumaroyl) rhamnoside was also from Persea lingue and was found to exert efflux inhibitory activity towards the NorA transporter of Staphylococcus aureus [34], whereas kaempferol 7-O-(2,3-di-E-p-coumaroyl-α-L-rhamnoside) was isolated from the flowers and fruit of Tetrapanax papyriferus [35]. The deprotonated molecular ion at *m*/*z* 753.1823 for compound **73** likely consisted of a derivative of the previous compound in which a feruloyl moiety was in the place of a p-coumaroyl residue. Finally, kaempferol di-p-coumaroylpentoside (**66**) showed the deprotonated molecular ion at *m*/*z* 709.1641 and TOF-MS/MS fragment ions at *m*/*z* 563.1244 (due to dehydrated p-coumaric acid), 423.1116 (coumaroylpentose-H_2_O), and 285.0416 (deprotonated kaempferol aglycone). Kaempferol 3-O-[2″,5″-di-O-(E)-p-coumaroyl]-α-L-arabinofuranoside was also isolated from Pseudotsuga menziesii [36]. Fatty acid derivatives, especially octadecanoids, accounted for the greater part of the tentatively identified compounds. In fact, beyond a dodecanedioic acid (**53**), likely traumatic acid, whose [M-H]^−^ ion fragmented gave the ions at *m*/*z* 183.1394 and 165.1278 by decarboxylation and following dehydration, respectively, and a dihydroxyhexadecanoic acid (**57**) with deprotonated molecular ion at *m*/*z* 287.2228, all the other fatty acids derivatives shared a carbon skeleton of 18 carbons.

Characteristic TOF-MS/MS fragment ions and peculiar neutral losses tentatively distinguished hydroxylation and unsaturation sites. The fragment ions at theoretical *m*/*z* 171.1027, 183.1027, and 183.1391, due to oxononanoate, oxodecenoate, and undecenoate, respectively, were mostly detectable. The octadecanoid 9,12,13-trihydroxy-10,15-octadecadienoic acid (**54**) was putatively identified. This compound, which showed the [M-H]^−^ ion at *m*/*z* 327.2191, was recently identified in aerial parts of Trifolium pratense [37]. The neutral loss of 98.0732 (theoretical value), likely due to hex-3-enal, provided the ion at *m*/*z* 229.1446, which dehydrated, giving the ion at *m*/*z* 211.1338 or, through CO loss, furnished the ion at *m*/*z* 183.1391. Compound **56** with deprotonated molecular ion at *m*/*z* 329.2337, based on the neutral loss of 100.0888 at the methyl end to achieve the ion at *m*/*z* 229.1448, was tentatively recognized as 9,12,13-trihydroxy-10-octadecenoic acid. This compound, herein identified for the first time in *F. sylvatica*, was firstly isolated from *Allium cepa*, and a prostaglandin E-like activity was observed [38]. Furthermore, its isolation was also from Panax quinquefolium [39]. Three isomers with the [M-H]^−^ ion at *m*/*z* 309.207, according to the C_18_H_30_O_4_ molecular formula, were also detected (compounds **58**, **59**, and **60**), and one of the hydroxyl functions was tentatively localized at C-16 carbon based on the neutral loss of 58.0419. This latter was identifiable also in compound **61** with the [M-H]^−^ ion at *m*/*z* 307.1915, suggesting that a dihydro derivative of the previous compounds occurred. Furthermore, two compounds with [M-H]^−^ ions at *m*/*z* 293.2122 (**75**) and 293.2121 (**79**), respectively, according to molecular formula C_18_H_30_O_3_, largely differing in their TOF-MS/MS fragment ions, were detected. TOF-MS/MS spectra of these compounds, beyond the common neutral loss of water, showed, in the first case, the ions at *m*/*z* 211.1340, 183.1392, and 171.1025, whereas the second appeared to be most sensitive to CO_2_ neutral loss, providing the ion at *m*/*z* 249.2220. In the TOF-MS/MS spectrum of the second compound, the ion at *m*/*z* 185.1179 was observed. A further unsaturated compound (**76**) was at *m*/*z* 291.1971 [40], which shared with the previous ones the loss of water and carbon dioxide to give the ions at *m*/*z* 273.1851 and 247.2069. The [M-H]^−^ ions at *m*/*z* 295.2283 and 295.2286 for compounds **77** and **78**, respectively, and at *m*/*z* 297.2444 for compound **80** were in accordance with the oxygenated derivatives of α-linolenic (**81** and **82**) and linoleic acid, respectively. Compound **79** was likely a dihydro derivative of compound **78**. These C-18 n:3 (**80**) and n:2 (**81**) were also detected, together with monounsaturated oleic acid (at *m*/*z* 281.2491; **84**) and the saturated fatty acids palmitic (at *m*/*z* 255.2336; **83**) and stearic acid (at *m*/*z* 283.2656; **85**).

### 2.2. Effects of Beech Leaf Alcoholic Extract and Its Fractions on Fermentative Parameters

The effect of the chemical composition of the beech Fs/1/1 extract and its Fs/2/1 and Fs/3/2 fractions was evaluated based on rumen fermentation parameters. These are attributable to the ecological microbiota community, which is, in turn, affected by different factors, such as animal age, breeding system, and diet and feeding technique. The effects of common beech are not available in the literature, even if plants such as *Quercus robur* L. [41,42] and *Castanea sativa* L. [43,44], both belonging to the *Fagaceae* family, were investigated. Odeyinka et al. [45] reported the effects of twelve Scottish plants, including the leaves of *F. sylvatica* L., on rumen fermentation. The authors observed a reduction in diet digestibility, suggesting the possible influence of the high phenolic content. The in vitro fermentation characteristics are listed in Table 2.

**Figure 5:**
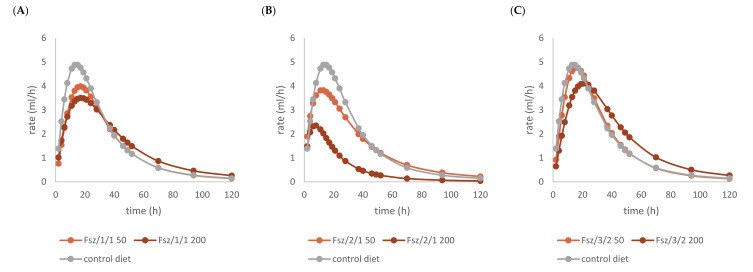
In vitro fermentation rate over time of *F. sylvatica* Fs/1/1 extract (**A**) and its fractions Fs/2/1 (**B**) and Fs/3/2 (**C**) at the 50-mg (●) and 200-mg (●) dose levels and control diet (●).

The in vitro gas production was investigated to preliminarily ascertain the fermentation features of common beech extract and its different chemically constituted fraction. For this purpose, the in vitro gas production technique (IVGPT) was applied. The IVGPT reproduces at the laboratory scale the feed degradation occurring in the rumen and studies the fermentation kinetics, also estimating its nutritional value [46]. The organic matter degradability (OMD) and gas produced after 120 h of incubation (OMCV and A) showed lower values for all the beech-based diet samples, except Fs/3/2 at the 200-mg dose level, than the control diet. The lipophilic fraction Fs/2/1 at the 200-mg dose level exhibited the lowest value: the fraction supplement was able to decrease by 1.7, 2.7, and 2.9-fold the OMD, OMCV, and A values, respectively. In vitro fermentation kinetics highlighted the peculiar behavior of the Fs/2/1 200-mg sample, which showed the lowest values for B, C, T_max_, and R_max_. The Fs/3/2 fraction, on the other hand, showed a dose-dependent increase in the B and T_max_ parameters, while a very weak decrease in R_max_ was dose-dependently observed. The differences between substrates in the fermentation process are clearer in Table 2B and Figure 5, where the gas production rate and in vitro fermentation rate over time are shown. The curve of the Fs/2/1 fraction at the 200-mg dose level reached rapidly the asymptote (T_max_: 7.39 and R_max_ 2.36; *p* < 0.01), showing a slowdown of the process after 40 h of incubation. Otherwise, the curve related to Fs/3/2 200 mg reached half of the asymptote later (B: 35.24 h; *p* < 0.01), and the fermentation process continued throughout the 120 h of incubation. The gas production kinetics obtained incubating the Fs/3/2 lowest dose appeared similar to that exhibited by the control diet. Data acquired in terms of the pH and concentration of the fermentation end products registered after 120 h of incubation are listed in Table 3. The rumen pH, which depends mainly on diet characteristics and physiological condition ranges between 6.2 and 7.5 [47], was not modified by the extract addition, and no significant difference was observed among the different extract concentrations. Oskoueian et al. [48], evaluating the effect of several flavonoids on the pH and ammonia nitrogen production, highlighted that the flavonoid addition did not alter these parameters. Accordingly, beech polyphenol containing extracts, such as Fs/1/1 and its fraction Fs/3/2, displayed similar ruminal pH to that exhibited by the control diet. Otherwise, the ruminal NH_3_-N production was significantly (*p* < 0.001) affected by the addition of beech extracts. NH_3_-N is a crude predictor of the efficiency of dietary N conversion into the microbial N total and a primary source of microbial growth [49]. Fraction Fs/2/1 slightly decreased the NH_3_-N concentration at both the tested concentrations, suggesting an interacting role of oxygenated unsaturated fatty acids with dietary proteins, which could reduce their utilization by the rumen microorganism. Moreover, the significant decrease of VFA production in both Fs/2/1 doses seems to confirm the detrimental effect of lipids of the rumen fermentative process [50]. 

The in vitro fermentation end products were reported as a proportion (%) of the single volatile fatty acids towards the total volatile fatty acids content (Table 4). Volatile fatty acids (VFAs), among which acetate, propionate, and butyrate are the major ones, provide approximatively 70% of the ruminant energy requirement. Acetate is used as primary energy source in the lipogenic process, whereas propionate is a gluconeogenesis precursor, and butyrate is mainly metabolized in D-3-hydroxybutyrate [51]. Other fatty acids of microbial origin are odd-chain and branched FAs (OBCFA), which could be found as milk constituents and utilized to predict volatile fatty acids production in the rumen [51,52]. The fraction Fs/3/2 positively affects VFAs production. These compounds, with adenosine triphosphate (ATP), CO_2_, H_2_, and CH_4_, and other minor compounds, are formed by the degradation of carbohydrates in the rumen.

The effect observed appeared to be dose-dependent. In fact, when the dose of 50 mg was considered, an increase equal to 12.3% was observed with respect to the control diet, whereas a VFAs content similar to the control diet was detected for the 200-mg dose. This finding is in line with the observation that dietary polyphenols could differently affect nutrient utilization efficiency based on the level of their inclusion in the diet and that high polyphenol doses alter the membrane permeability [53]. Analogously, the dose level massively impacted the total volatile fatty acids amount, according to the double-edge sword role of polyphenol compounds in redox status maintenance and their antimicrobic efficacy [54]. In fact, the Fs/3/2 fraction at the 50-mg dose level was able to increase the three main VFAs absorbed from the rumen: acetic, propionic, and butyric acids by 5.0, 34.4, and 36.8%, respectively, while a lower increase of butyric acid concentration (13%) was observed in the diet enriched with the Fs/3/2 fraction at the 200-mg dose level.

The influence of the Fs/3/2 fraction dose level on rumen microbiota [55] in terms of population or activity was suggested also by the calculated A:P ratios, which were found equal to 2.32 and 3.20 for the low-dose and high-dose treatments, respectively. Thus, the low dose of the polyphenol beech fraction, by contrast with its high dose, was able to reduce the ruminal acetate-to-propionate ratio with respect to the control diet, improving the diet utilization efficiency. The total VFA was positively correlated to the low dose of Fs/3/2 with the highest content of total phenolic (r = 0.983) and flavonoid amount (r = 0.986) (Figure S2), according to previous findings. In fact, Acacia nilotica leaf exhibited a higher substrate degradability, total volatile fatty acid, mainly propionic acid, production, and lower methanogenesis [56]. Analogously, mulberry leaf, or different phenolic compounds from propolis extract, showed an increase of total volatile fatty acids and acetate, butyrate, and propionate [57,58]. Several reports have illustrated that flavonoids may regulate ruminal microbiota and that different classes of flavonoids have distinct effects. Similarly, the ruminal biomass can be influenced by in vitro incubation with different plant-derived substrates [59,60]. The increase of iso-butyric and iso-valeric acids was also observed in the Fs/3/2 50-mg diet sample. This could be due to the possible increase of protein digestibility and/or may to changes in the composition of the rumen bacteria population [61]. In fact, isobutyric and isovaleric acids are synthesized by rumen microorganisms via an oxidative deamination and an oxidative decarboxylation starting from the branched-chain amino acids, valine, and leucine, respectively [47]. Additionally, other studies observed that flavonoids, pure [48,62] or in a mixture [63], are able to improve VFAs production; the flavanone naringenin appeared to mainly increase acetate [48]. 

The impact of the Fs/2/1 diet samples had a different behavior as a marked decrease of total VFA was measured for both the dose-level treatments considered. In particular, butyric acid, which is a signaling molecule that indirectly stimulates epithelial proliferation in growing calves before weaning [64], was slowed down by 37% in the Fs/2/1 200-mg diet sample. Propionic acid appeared to be massively decreased following both the treatments, so much so that a percentage decrease by 6.2% was observed in the Fs/2/1 50-mg diet sample and by 31.7% in the Fs/2/1 200-mg diet sample. In this context, the A:P ratios appeared to weakly augment a peculiar increase in BCFA following the Fs/2/1 diet sample treatments, up to an increase of over 80% with respect to the control diet for the 200-mg dose level. Contrariwise, at the same dose level, the polyphenol fraction reduced BCFA by 30.7%, while the parental extract, which also accounted in large part for the hydroxycinnamoyl and flavonoid compounds, resulted in a reduction of 10%.

Thus, fractions from the beech alcoholic extract could represent alternatives, differently efficacious, for feeding livestock. This is in line with the key issue for the sustainable development of animal production, which lays its foundation on the efficient use of resources with a reducing waste. This aspect, coupled with the high costs of feedstuffs and the increasing demand for dairy products for human consumption, necessarily involves the research for new alternatives of feeding livestock, able to positively affect animals’ health and performance. Beech fractions exert different antiradical and reducing activities and differently affect the rumen fluid fermentation parameters.

## 3. Materials and Methods

### 3.1. Plant Collection and Fractionation

The leaves of *F. sylvatica* L. were collected in June 2017 in the Tannenberger Gehölz National Forest area (Kiel, Schleswig-Holstein, Germany, N 54°21′52.6″, E 10°06′35.9″, 25 m a. m. s. l., Google Earth). Voucher specimens were deposited in the herbarium of Kiel University (KIEL) and the private herbarium of Christian Zidorn (voucher code: FS_20160705A-1). The leaves were first air-dried and minced and then ultrasound-assisted macerated (Branson Ultrasonics^TM^ Bransonic^TM^ M3800-E, Danbury, CT, USA) using methanol (leaves/solvent ratio 1:8, g:mL) at room temperature (set at 25 °C). Three sonication cycles were carried out, each one of 30 min, for obtaining the Fs/1/1 extract. The extract yield (%) was equal to 14.8% (45.5 g). The alcoholic extract was then dissolved in a biphasic solution CHCl_3_:MeOH:H_2_O (13:7:6, *v*:*v*:*v*), and discontinuous liquid–liquid extraction (LLE) was performed. Thus, an organic fraction (Fs/2/1; yield 31.2% of Fs/1/1) and a hydroalcoholic one (Fs/2/2) were obtained. The fraction Fs/2/2 was chromatographed on XAD-4 resin using water first and then methanol. The alcoholic fraction Fs/3/2 was obtained with a yield equal to 12.4%.

### 3.2. UHPLC-HRMS and MS/MS Parameters and UV-Vis Analyses

Fs/1/1 extract and the fractions therefrom were first analyzed by UV–Vis spectrophotometry in the range 200–800 nm by a Cary 100 spectrophotometer. The three samples (10 mg/mL) were then profiled by a Shimadzu NEXERA UHPLC system (Shimadzu, Tokyo, Japan). A Luna^®^ Omega C18 (1.6-μm particle size, 50 × 2.1 mm i.d.) was utilized, and 2.0 μL of each sample were injected. The separation was achieved using a binary solution: (A) H_2_O (0.1% HCOOH) and (B) CH_3_CN (0.1% HCOOH). A linear gradient was used in which the percentage of solvent B increased as follows: 0–5 min, 5%→12% B; 5–13 min, 12%→22% B; 13–23 min, 22%→45% B; 23–26 min, 45%→65% B; 26–30 min, 65%→95%; 30–33 min, 95% B; and 33.01–35 min, column re-equilibration. The flow rate was set at 400 μL/min. MS analysis was performed using a hybrid Q-TOF MS instrument, the AB Sciex Triple TOF^®^ 4600 (AB Sciex, Concord, ON, Canada), equipped with a DuoSpray^TM^ ion source, which was operated in the negative ElectroSpray (ESI) mode. The APCI probe was used for automated mass calibration using the Calibration Delivery System. A full-scan time-of-flight (TOF) survey (dwell time 250 ms, 100–1500 Da) and eight IDA MS/MS scans (dwell time 100 ms, 80–1300 Da) were acquired, using the following parameters: curtain gas (CUR) 35 psi, nebulizer (GS1) and heated (GS2) gases 60 psi, ion spray voltage (ISVF) 4500 V, ion source temperature (TEM) 600 °C, and declustering potential (DP) −70 V. The collision energy (CE) applied was −35 V, with a collision energy spread (CES) of 15 V. The instrument was controlled by Analyst^®^ TF 1.7 software (AB Sciex, Concord, ON, Canada, 2016), while data processing was carried out using PeakView^®^ software version 2.2 (AB Sciex, Concord, ON, Canada, 2016).

### 3.3. Radical Scavenging Capacity: DPPH and ABTS Tests

The leaf extract and fractions therefrom were tested at 200, 100, 50, 25, 12.5, 6.25, and 3.125 μg/mL (final concentration levels) towards ABTS (2,2′-azinobis-(3-ethylbenzothiazolin-6-sulfonic acid)) radical cation and 2,2-diphenyl-1-picrylhydrazyl (DPPH) radical. Trolox (4, 8, 16, and 32 µM) was used as the standard, and all recorded activities were compared to a blank sample, arranged in parallel. ABTS^●+^ was generated by mixing (2,2′-azinobis-(3-ethylbenzothiazolin-6-sulfonic acid); 7 mM) and potassium persulfate (K_2_S_2_O_8_; 2.45 mM), in the dark for 12 h. ABTS^●+^ was diluted with PBS (pH 7.4) until an absorbance equal to 0.7 at 734 nm was reached. Thus, the extract doses were dissolved in ABTS^●+^ solution, and after 6 min, the absorbance was measured using a Wallac Victor3 spectrophotometer in reference to a blank [65]. DPPH^●^ methanol solution (9.4 × 10^−5^ M) and extract doses were utilized to assess the DPPH^●^ scavenging capability. The mixtures were stirred for 15 min, and the absorption was read at 517 nm by a Wallac Victor3 spectrophotometer in reference to a blank. The results were expressed in terms of the percentage reduction of the initial radical adsorption by the tested samples [66]. Trolox (4, 8, 16, and 32 µM) was used as the positive standard. All data were expressed as the mean ± standard deviation (SD).

### 3.4. Fe (III) Reducing Power

The ability to reduce the Fe(III) of beach leaf Fs/1/1 extract and its fractions (at the 200,100, 50, 25, 12.5, 6.25, and 3.125-µg/mL final concentration levels) was evaluated using the ferricyanide FRAP assay, according to PFRAP procedure [25]. The absorbance was measured at 700 nm. The increase in absorbance with reference to the blank was considered. Trolox (4, 8, 16, and 32 µM) was used as the positive standard. All data were expressed as the mean ± standard deviation (SD).

### 3.5. Determination of Total Phenolic Content

The total phenolic content was determined according to the Folin–Ciocalteu procedure [65]. Aliquots of the samples (0.25 mg and 0.125 mg) were mixed with 2.25 mL of Na_2_CO_3_ (7.5% *w*/*v*) and 0.25 mL of Folin–Ciocalteu reagent. The tubes were mixed and allowed to stand for 3 h at room temperature (T = 25 °C). The absorbance was read at 765 nm using a Synergy Biotek spectrophotometer. The total phenol content was expressed as milligrams of gallic acid equivalents (GAEs) per g of extract.

### 3.6. Determination of Total Flavonoidic Content

NaNO_2_ (5%, *w*/*v*; 0.3 mL) was added to the samples (1 mg and 2 mg), firstly solubilized into 5 mL of distillate water. After 10 min, AlCl_3_ (10%, *w*/*v*; 0.6 mL) was added. The reaction was carried out for 6 min. Then, NaOH (1.0 M, 2.0 mL) was added, and the mixture was diluted to 10 mL with distillate water. The absorbance was read at 510 nm against the blank (water) using a Synergy HT Biotek spectrophotometer. The flavonoid content was expressed as milligrams of quercetin equivalents (QUEs) per g of extract [67].

### 3.7. Determination of Total Lipidic Content

Beech samples (at 0.5 mg, 0.25 mg, and 0.125 mg) were mixed with 150 µL of isovanillin/glacial acetic acid (5% *w*/*v*) and 500 µL of oxidant perchloric acid [68]. The mixture was incubated at 60 °C for 45 min and cooled down in an ice bath. Ethyl acetate was added in order to obtain 2 mL as the total volume. Absorbance was read using a Synergy HT Biotek spectrophotometer at 550 nm. The total saponins were quantified by using a standard curve of calibration of oleanolic acid and expressed as milligrams of oleanolic acid equivalents (OAEs) per g of extract.

### 3.8. In Vitro Fermentation

Beech Fs/1/1 extract and its Fs/2/1 and Fs/3/2 fractions were incubated at 0 (control), 50, and 200-mg dose levels with one gram of a diet composed by mixed hay, corn silage, and concentrate (crude protein: 15.0%, NDF: 46.0%) in hermetically sealed serum flasks (120 mL each, three replications for each treatment) with buffered rumen fluid (10 mL) at 39 °C under anaerobic conditions [46]. The rumen liquor was collected at a slaughterhouse [69] from four buffalos fed a total mixed ratio. All procedures involving animals were approved by the Ethical Animal Care and Use Committee of the University of Naples Federico II (Prot. 2019/0013729 on 08/02/2019). The gas produced during the 120 h of incubation into the fermenting flasks was recorded using a manual pressure transducer (Cole and Palmer Instrument Co, Vernon Hills, IL, USA) and related to incubated OM (OMCV, mL/g). At the end of the incubation period, the pH of the fermentation liquor was measured by a pH meter (ThermoOrion 720 A+, Fort Collins, CO, USA). The organic matter degradability (OMD, %) was assessed by weight differences of the incubated OM, and the undegraded filtered (sintered glass crucibles; Schott Duran, Mainz, Germany, porosity # 2) residue burned at 550 °C for 3 h.

### 3.9. Fermentation End Products Assessment

To determine the volatile fatty acids (VFAs, mmol/g) production, the fermentation liquor was first cooled at 4 °C and centrifuged at 12,000× *g* for 10 min (Universal 32R centrifuge, Hettich FurnTech Division DIY, Melle-Neuenkirchen, Germany). The supernatant (1 mL) was then mixed with oxalic acid (1 mL; 0.06 mol). The VFA was measured by gas chromatography (ThermoQuest 8000top Italia SpA, Rodano, Milan, Italy) equipped with a fused silica capillary column (30 m, 0.25 mm ID, 0.25-μm film thickness). Quantitation was based on an external standard solution of acetic, propionic, butyric, iso-butyric, valeric, and iso-valeric acids. Branched-chain fatty acids (BCFAs) percentages were calculated as follows: (iso-butyric acid +iso-valeric acid/tVFAs)/100. The ammonia nitrogen (N-NH_3_, mmol/g) production was colorimetrically assessed [70].

### 3.10. Data Processing and Statistical Analysis

Colorimetric tests were carried out performing three replicate measurements for three samples (*n* = 3) of the extract (in total, 3 × 3 measurements). All data were expressed as the mean ± standard deviation (SD).

To estimate the fermentation kinetic parameters, the gas production profiles were fitted to the sigmoidal model [71]: G = A/(1 + B/t)C, where G is the total gas produced (mL/g of incubated OM) at time t (h), *A* is the asymptotic gas production (mL/g), *B* is the time at which one-half of *A* is reached (h), and *C* is the curve switch. The maximum fermentation rate (*R_max_*, mL/h) and the time at which it occurs (*T_max_*, h) were calculated utilizing the formula suggested by Bauer et al. [72]:

$$R_{max}=\frac{\left(A\times C^{B}\right)\times B\times T_{max}^{\left(B-1\right)}}{\left(1+C^{B}\right)\times {\left(T_{max}^{-B}\right)}^{2}}$$

$$T_{max}=C\;\times \;{\left(\frac{B-1}{B+1}\right)}^{1/B}$$

Statical analyses were performed by ANOVA for one-way using software SigmaPlot 14.0 to evaluate the substrate effect. The in vitro parameters (OMCV, OMD, *A*, *B*, *C*, *T_max_*, and *R_max_*) and the end products data (pH, N-NH_3_, VFA, and BCFA) were statistically analyzed, and the significant levels were verified using HSD Tukey’s test at *p* < 0.05, *p* < 0.01, and *p* < 0.001. The correlations between colorimetric assays values and fermentation parameters were also evaluated using Pearson’s correlation coefficient (JASP 14.0; Figures S2 and S3).

## 4. Conclusions

Plant materials with high nutrient contents and elevated digestibility can be used as a supplementary resource for the feeding of small and large ruminants. When used in animal nutrition, byproducts of biorefining of a forest biomass will provide bioactive compounds that could modulate rumen microbes with beneficial effects for the environment or increasing the nutraceutical value of human food from animal sources (e.g., fatty acids, antioxidants, etc.). Herein, beech leaves appear as a promising material affecting ruminal fermentation, and the chemical composition of the beech prepared extract/fraction is the main actor in the recorded effects. In particular, it was observed that the addition of 50 mg of the polyphenols-rich fraction (named Fs/3/2) to a ruminant diet significantly (*p* < 0.05) increased the production of the total VFAs and the relative contents of acetic (*p* < 0.05), propionic (*p* < 0.001), and butyric acids, also reducing the gas production and fermentation rate. The data acquired are a starting point for further research, aimed at exploring the in vivo feasibility of *F. sylvatica* L. leaf extract/fraction as supplements in ruminant nutrition. The dose–response efficacy of the beech extract/fraction further emphasizes the need for future investigations.

## Figures and Tables

**Table 1:** Metabolites tentatively identified in the beech Fs/1/1 alcoholic extract and its Fs/2/1 and Fs/3/2 fractions. RT = retention time; RDB = ring double bond equivalent value. Base peak fragments are reported in bold. 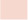 detected.

Peak	Rt (min)	Tentative Assignment	Formula	[M-H]− Found (*m*/*z*)	[M-H]− Calc. (*m*/*z*)	Error (ppm)	RDB	MS/MS Fragment Ions (*m*/*z*) and Relative Intensity	Fs/1/1	Fs/2/1	Fs/3/2
1	0.397	Quinic acid	C_7_H_12_O_6_	191.0557	191.0561	−2.2	2	191.0557;129.0192; 111.0096; **87.0098**; 85.0307			
2	0.454	Citric acid	C_6_H_8_O_7_	191.0203	191.0197	3	3	111.0089; **87.0089**			
3	0.769	Galloyl hexose	C_13_H_16_O_10_	331.0667	331.0671	−1.1	6	331.0662; 211.0236; **169.0139**; 151.0033			
4	1.311	Dihydroxybenzoic acid hexoside	C_13_H_16_O_9_	315.0720	315.719	0.1	6	315.0722, 271.0396, 227.0528, 195.0287, 153.0199; **152.0114**			
5	1.574	Hydroxybenzoyl hexose	C_13_H_16_O_8_	299.0770	299.0772	−0.8	6	299.0770; 239.0557; 179.0345; 151.0394; **137.0237**			
6	1.890	Hydroxyphenylacetic acid hexoside	C_14_H_18_O_8_	313.0939	313.0929	3.2	6	313.0910; **151.0390**			
7	2.038	3-O-Caffeoyl quinic acid (cis)	C_16_H_18_O_9_	353.0885	353.0878	2	8	353.0888; **191.0565**; 179.0353;135.0451			
8	2.235	3-O-Caffeoyl quinic acid (trans)	C_16_H_18_O_9_	353.0888	353.0878	2.2	8	353.0888; **191.0565**; 179.0353;135.0451			
9	2.444	Caffeoyl threonic acid (1)	C_13_H_14_O_8_	297.0614	297.0622	2	7	179.0340; 161.0245; **135.0310**; 117.0192; 89.0248			
10	2.603	Caffeoyl acid hexoside	C_15_H_18_O_9_	341.0874	341.0878	7	−1.2	179.0344; **161.0244**; 135.0444; 134.0296			
11	2.822	Caffeoyl threonic acid (2)	C_13_H_14_O_8_	297.0618	297.0616	0.7	7	179.0344; **135.0301**			
12	2.839	3-O-*p*-Coumaroyl quinic acid	C_16_H_18_O_8_	337.0926	337.0929	−0.9	8	**191.0560**; 163.0401; 119.0499			
13	3.210	3-O-*p*-Coumaroyl quinic acid	C_16_H_18_O_8_	337.0926	337.0929	−0.9	8	191.0556; **163.0340**; 119.0502			
14	3.444	Procyanidin	C_30_H_26_O_12_	577.1353	577.1352	0.3	18	577.1393, 451.1046, 425.0883, **407.0781**, 289.0711, 245.0463, 125.0236			
15	3.523	*p*-Coumaroyl acid hexoside	C_15_H_18_O_8_	325.0926	325.0929	−0.9	7	163.0393; **119.0500**			
16	3.676	*p*-Coumaroyl threonic acid (1)	C_13_H_14_O_7_	281.0662	281.0666	−1.7	7	163.0396; 135.0298; **119.0502**			
17	3.950	Caffeic acid	C_9_H_8_O_4_	179.0357	179.0350	4.0	6	**135.0454**; 134.0377;117.0348; 107.0508; 89.0403			
18	4.259	*p*-Coumaroyl threonic acid (2)	C_13_H_14_O_7_	281.0666	281.0666	0	7	177.0570; 163.0399; 145.0293; 135.0298; **119.0503**; 117.0196; 87.0089			
19	4.624	5-*O*-Caffeoyl quinic acid (trans)	C_16_H_18_O_9_	353.0879	353.0878	0.3	8	**191.0571**; 85.0306			
20	5.665	Caffeoyl threonic acid (3)	C_13_H_14_O_8_	297.0616	297.0612	−1.3	7	179.0343; 161.0242; **135.0242**			
21	5.693	Caffeoyl propionic acid	C_12_H_12_O_6_	253.0718	253.0718	0.2	6	253.0705; 179.0344; **161.0243**; 135.0450; 134.0375; 133.0375			
22	5.759	5-*O*-Caffeoyl quinic acid (cis)	C_16_H_18_O_9_	353.0874	353.0878	−1.1	8	**191.0557**; 161.0240;85.0293			
23	6.090	Eriodictyol 7-*O*-hexoside	C_21_H_22_O_11_	449.1092	449.1089	−0.3	11	449.1093; 421.11444; 313.0705; 287.0553; 283.0602; **259.0607**; 243.0665; 215.0702; 178.9980; 125.0242			
24	6.645	Tuberonic acid	C_18_H_28_O_9_	387.1662	387.1661	0.4	5	**387.1655**; 207.1024; 163.1130; 89.0243			
25	6.981	5-*O*-*p*-Coumaroyl quinic acid	C_16_H_18_O_8_	337.0930	337.0929	0.3	8	**191.0559**			
26	7.312	*p*-*O*-Coumaroylmalic acid (I)	C_13_H_12_O_7_	279.0508	279.0510	−0.1	8	279.0506; 179.0352; **161.0248**; 133.0301			
27	7.871	Caffeoylshikimic Acid	C_16_H_16_O_8_	335.0766	335.0772	−1.9	9	335.0766; 179.0355; **161.0246**; 135.0450			
28	8.101	5-*O*-*p*-Coumaroyl quinic acid	C_16_H_18_O_8_	337.0925	337.0929	−1.2	8	**191.0560**; 173.0461; 93.0347; 85.0296			
29	8.314	Caffeoylshikimic Acid	C_16_H_16_O_8_	335.0770	335.0772	−0.7	9	**179.0350**; 161.0243; 135.0453			
30	9.288	*p*-*O*-Coumaroylmalic acid (II)	C_13_H_12_O_7_	279.0508	279.0510	−0.1	8	179.0348; **135.0451**			
31	10.091	Naringenin-*C*-hexoside (1)	C_21_H_22_O_10_	433.1145	433.1140	1.1	11	433.1153; 415.1027; 343.0826; 325.0715; **313.0715**; 271.0611; 223.0245; 193.0143; 119.0506			
32	10.712	Naringenin-*C*-hexoside (2)	C_21_H_22_O_10_	433.1154	433.1140	1.6	11	433.1151; 415.1043; 343.0826; 325.0710; **313.0718**; 283.0610; 271.0611; 223.0246; 193.0142; 151.0040			
33	11.960	Myricetin-3-*O*-hexoside	C_21_H_20_O_13_	479.0839	479.0831	1.6	12	479.0841; 317.0286; **316.0221**; 287.0193; 271.0239			
34	14.293	Quercetin-3-*O*-hexoside (1)	C_21_H_20_O_12_	463.0893	463.0882	2.4	12	463.0898; 301.0355; **300.0270**; 271.0246; 255.0298; 178.9989; 151.0035			
35	14.445	Quercetin-3-*O*-hexuronide	C_21_H_18_O_13_	477.0679	477.0675	0.9	13	477.0688; **301.0347**; 178.9979; 151.0033			
36	14.728	Quercetin-3-*O*-hexoside (2)	C_21_H_20_O_12_	463.0891	463.0882	1.9	12	463.0899; 301.0355; **300.0271**; 271.0247; 255.0295; 243.0296; 178.9985; 151.0035			
37	14.739	Kaempferol-3-*O*-hexuronide (1)	C_21_H_18_O_12_	461.0733	461.0725	1.6	13	**285.0398**			
38	14.940	Naringenin-*C*-hexoside (3)	C_21_H_22_O_10_	433.1145	433.1140	1.1	11	433.1146; 415.1026; 343.0827; 325.0731; **313.0713**; 283.0602; 271.0605; 223.0236; 193.0138; 119.0503			
39	15.083	Neolignan (I)	C_25_H_34_O_11_	509.2033	509.2028	0.9	9	509.2033; 491.1937; 461.1823; 367.1396; 313.1288; **179.0711**; 167.0708; 149.0605; 147.0446; 134.0372			
40	15.218	Neolignan (II)	C_25_H_34_O_11_	509.2043	509.2028	2.9	9	509.2052; 491.1949; 473.1834: 461.1832; 367.1406; 313.1301; **179.0717**; 149.0608			
41	15.338	Quercetin-3-*O*-pentoside	C_20_H_18_O_11_	433.0783	433.0776	1.5	12	433.0795; 301.0359; **300.0277**; 271.0250; 255.0299; 243.0294; 178.9986			
42	15.423	Kaempferol-3-*O*-hexoside (1)	C_21_H_20_O_11_	447.0944	447.0933	2.5	12	447.0945; 285.0398; **284.0318**; 255.0292; 227.0340; 151.0031			
43	15.517	Quercetin-3-*O*-(acetyl)hexoside	C_23_H_22_O_13_	505.1003	505.0988	3.0	13	505.1018; 463; 0897; 447.0945; 301.0357; **300.0277**; 271.0238; 255.0296			
44	15.743	Kaempferol-3-*O*-hexuronide (2)	C_21_H_18_O_12_	461.0741	461.0725	3.4	13	461.0739; **285.0406**; 257.0460; 229.0511; 113.0248			
45	15.903	Kaempferol-3-*O*-hexoside (2)	C_21_H_20_O_11_	447.0951	447.0933	4.1	12	447.0944; 327.0504; 285.0397; **284.0317**; 255.0295; 227.0345; 151.0040			
46	16.237	Kaempferol-3-*O*-pentoside (1)	C_20_H_18_O_10_	417.0843	417.0827	3.8	12	417.0836; 285.0399; **284.0323**; 255.0295; 227.0345; 151.0036			
47	16.456	Kaempferol-3-*O*-pentoside (2)	C_20_H_18_O_10_	417.0833	417.0827	1.4	12	417.0841; 285.0406; **284.0330**; 255.0300; 227.0349			
48	16.748	Kaempferol-3-*O*-(acetyl)hexoside	C_23_H_22_O_12_	489.1049	489.1039	2.1	13	489.1069; 429.0800; 369.0996; **285.0412**; 284.0333; 255.0301; 227.0350; 151.0034			
49	17.073	Kaempferol-3-*O*-deoxyhexoside	C_21_H_20_O_10_	431.0991	431.0984	1.7	12	431.1002; 285.0408; **284.0332**; 255.0303; 227.0352; 229.0506			
50	18.232	Neolignan (III)	C_27_H_38_O_12_	553.2313	553.2291	4.1	9	553.2330; **343.1403**; 328.1162; 183.0655			
51	19.380	Kaempferol *p*-coumaroylhexoside	C_30_H_26_O_13_	593.1325	593.1320	3.3	18	593.1322; 447.0935; 307.0813; **285.0387**; 284.0323; 255.0294			
52	19.623	Kaempferol	C_15_H_10_O_6_	285.0404	285.0405	−0.2	11	**285.0400**; 257.0445; 255.0293; 229.0495; 211.0393; 187.0393; 151.0034			
53	18.438	Dodecenedioic acid	C_12_H_20_O_4_	227.1289	227.1300	4.0	3	**183.1394**; 165.1278			
54	18.867	9,12,13-trihydroxy-10,15-octadecadienoic acid	C_18_H_32_O_5_	327.2191	327.2177	0.9	3	309.2076; 291.1970; 239.1288; 229.1446; 221.1179; **211.1338**; 191.1236; 183.1391; 171.1022; 137.0966			
55	21.080	Neolignan (IV)	C_38_H_52_O_16_	763.3187	763.3183	0.6	13	763.3225; **343.1403**; 328.1154; 183.0654			
56	19.695	9,12,13-trihydroxy-10-octadecenoic acid	C_18_H_34_O_5_	329.2337	329.2340	2.0	2	329.2343; 229.1448; **211.1344**; 183.1393; 171.1030			
57	20.297	Dihydroxyhexadecanoic acid	C_16_H_32_O_4_	287.2228	287.2240	4.2	1	**287.2235**; 269.2127			
58	20.558	9,16-diidrossi-octadeca-10, 12,14-trienoic acid (1)	C_18_H_30_O_4_	309.2072	309.2071	0.2	4	309.2078; 291.1972; 273.1841; 251.1654; 239.1650; 221.1544; 197.1162; 183.1026; **171.1032**; 107.0865			
59	20.846	16-idrossi-9-ossooctadeca-12,14-dienoic acid	C_18_H_30_O_4_	309.2071	309.2071	−0.1	4	309.2059; 291.1962; **251.1655**; 171.1028; 125.0971			
60	21.053	9,16-diidrossi-octadeca-10, 12,14-trienoic acid (2)	C_18_H_30_O_4_	309.2071	309.2071	0.2	4	309.2079; **291.1967**; 251.1649; 185.1180; 171.1021; 137.0971			
61	21.350	16-idrossi-9-ossooctadeca-6,12,14-trienoic acid	C_18_H_28_O_4_	307.1915	307.1925	3.3	5	289.1793; 249.1492; 235.1342; 211.1340; 185.1187; 125.0976; **121.0659**			
62	21.350	Dihydroxyoctadecenoic quinic acid	C_25_H_42_O_9_	485.2776	485.2756	4.1	5	485.2791; **311.2238**; 223.1706; 191.0568			
63	22.220	16-idrossi-9-ossooctadeca-6,12,14,16-tetraenoic acid	C_18_H_26_O_4_	305.1758	305.1760	0.5	6	305.1744; 249.1498; 205.1587; **135.0817**			
64	22.435	Kaempferol *p*-coumaroyldeoxyhexoside (1)	C_30_H_26_O_12_	577.1368	577.1352	2.9	18	577.1389; 431.0988; **285.0404**; 284.0316; 257.0452; 299.0495			
65	22.553	Kaempferol *p*-coumaroyldeoxyhexoside (2)	C_30_H_26_O_12_	577.1357	577.1352	1.0	18	577.1382; **285.0399**; 284.0328			
66	22.842	Kaempferol di-*p*-coumaroylpentoside	C_38_H_30_O_14_	709.1463	709.1590	3.8	24	709.1641; 563.1244; 423.1116; **285.0416**; 284.0332; 145.0290			
67	23.154	15,16-dihydroxy-9Z,12Z- octadecadienoic acid	C_18_H_32_O_4_	311.2242	311.2228	1	3	311.2238; 293.2112; 275.2011; 235.1708; **223.1705**; 201.1135; 87.0454			
68	23.310	3-*O*-(dihexosyl)hexuronidyl oleanonic acid	C_48_H_76_O_19_	955.4928	955.4908	2.1	11	**955.4984**; 793.4427; 731.4414; 613.3786; 569.3871; 523.3830			
69	23.582	3-*O*-(dihexosyl)hexuronidyl oleanonic acid	C_48_H_76_O_19_	955.4923	955.4908	1.6	11	**955.4972**; 793.4423; 569.3868			
70	24.029	Dihydroxyoctadecenoic acid	C_18_H_34_O_4_	313.2382	313.2384	−0.7	2	313.2377; 295.2270; 277.2163; **183.1384**; 129.0916			
71	24.050	3-*O*-(hexosylpentosyl)hexuronidyl oleanonic acid	C_47_H_74_O_18_	925.4801	925.4802	−0.2	11	**925.4840**; 763.4277			
72	24.128	3-*O*-(hexosyl)hexuronidyl oleanonic acid	C_42_H_66_O_14_	793.4395	793.4380	0.9	10	**793.4407**; 631.3873; 569.3854			
73	24.457	Kaempferol di-*p*-coumaroyldeoxyhexoside	C_39_H_32_O_14_	723.1720	723.1719	0.1	24	**723.1784**; 577.1396; 559.1250; 437.1265; 285.0409; 284.0332; 187.0391; 163.0401; 145.0294			
74	24.483	Kaempferol *p*-coumaroyl feruloyl pentoside	C_40_H_34_O_15_	753.1825	753.1823	−0.3	24	753.1884; 607.1502; 589.1358; 467.1378; **285.0407**; 284.0320; 193.0495			
75	25.032	9-oxooctadeca-10,12-dienoic acid	C_18_H_30_O_3_	293.2122	293.2130	2.7	4	293.2126; **275.2019**; 235.1708; 211.1340; 183.1392; 171.1025; 121.1026			
76	25.302	9-oxooctadeca-10,12,15-trienoic acid	C_18_H_28_O_3_	291.1971	291.1966	1.8	5	**291.1969**; 273.1851; 247.2069; 223.1701; 195.1382			
77	25.611	13-hydroxyoctadeca-9,15-dienoic acid	C_18_H_32_O_3_	295.2283	295.2279	1.5	3	295.2284; 277.2176; **183.1389**			
78	25.783	13-hydroxyoctadeca-9,11-dienoic acid	C_18_H_32_O_3_	295.2286	295.2279	1.8	3	**295.2272**; 277.2166; 195.1387; 183.1023; 155.1065; 113.0971			
79	25.937	13-oxo-9Z,11E-octadecadienoic acid	C_18_H_30_O_3_	293.2121	293.2122	2.0	4	**293.2119**; 275.2022; 249.2220; 195.1390; 185.1179; 153.1287; 113.0974			
80	26.267	9-hydroxyoctadec-12-enoic acid	C_18_H_34_O_3_	297.2444	297.2435	3.0	2	**297.2439**; 279.2331; 171.1027; 155.1075			
81	27.520	α-Linolenic acid	C_18_H_30_O_2_	277.2180	277.2173	2.5	4	**277.2181**			
82	28.089	Linoleic acid	C_18_H_32_O_3_	279.2338	279.2330	3.0	3	**279.2347**; 259.2098			
83	28.552	Palmitic acid	C_16_H_32_O_2_	255.2336	255.2330	2.5	1	**255.2334**; 237.2220; 201.8341			
84	28.638	Oleic acid	C_18_H_34_O_2_	281.2491	281.2486	1.8	2	**281.2493**			
85	29.311	Stearic acid	C_18_H_36_O_2_	283.2656	283.2643	4.8	1	**283.2652**; 265.2517			

**Table 2:** (**A**) Cumulative gas production, organic matter degradability, and fermentation kinetics parameters of different *F. sylvatica* extracts. OMD: organic matter degradability; OMCV: cumulative volume of gas related to incubated organic matter. (A) Asymptotic gas production, (B) time at which one-half of the asymptote is reached, and (C) switching characteristics of the curve. R_max_: maximum fermentation rate; T_max_: time at which R_max_ occurs. * *p* < 0.01 and ** *p* < 0.001. (**B**) In vitro gas production over time of *F. sylvatica* Fs/1/1 extract and its fractions Fs/2/1 and Fs/3/2 at 50-mg (●) and 200-mg (●) dose levels and control diet (●).

**(A)**				**(B)**
**Parameter**	**control diet**	**Fs/1/1**		
		50 mg	200 mg	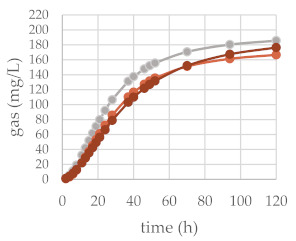
**OMD (%)**	77.43	75.96	71.98	
**OMCV (mL/g)**	225.59	210.02	216.75	
**A (mL/g)**	194.83	175.61 *	195.34	
**B (h)**	25.42	28.64	34.80 **	
**C**	1.93	2.04	1.80	
**T_max_ (h)**	14.37	16.88	17.34	
**R_max_ (mL/h)**	4.87	4.01	3.62	
	**control diet**	**Fs/2/1**		
		50 mg	200 mg	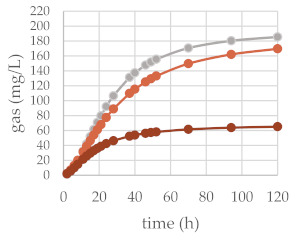
**OMD (%)**	77.43	74.01	45.37 **	
**OMCV (mL/g)**	225.59	212.13	83.37 **	
**A (mL/g)**	194.83	187.71	67.91 **	
**B (h)**	25.42	29.93	17.64 **	
**C**	1.93	1.61 **	1.64 *	
**T_max_ (h)**	14.37	13.47 **	7.39 **	
**R_max_ (mL/h)**	4.87	4.58	2.36 **	
	**control diet**	**Fs/3/2**		
		50 mg	200 mg	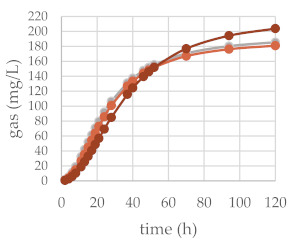
**OMD (%)**	77.43	76.63	74.40	
**OMCV (mL/g)**	225.59	217.17	241.98	
**A (mL/g)**	194.83	188.47	220.53 **	
**B (h)**	25.42	26.19	35.24 **	
**C**	1.93	2.08	2.04	
**T_max_ (h)**	14.37	15.83	20.82	
**R_max_ (mL/h)**	4.87	4.77	4.12	

**Table 3:** Effects of *F. sylvatica* L. extracts at different doses (50 mg and 200 mg) on fermentation end products after 120 h of incubation. N-NH_3_: ammonia-N; Total VFAs: total volatile fatty acid (acetate + propionate + butyrate + iso-butyrate + valerate + iso-valerate); BCFA: branched-chain fatty acid proportion (iso-butyrate + iso-valerate)/tVFA; A/P: acetate/propionate ratio. * *p* < 0.01, ** *p* < 0.001, and ǂ *p* < 0.05.

	Control Diet	Fs/1/1		Fs/2/1		Fs/3/2	
		50 mg	200 mg	50 mg	200 mg	50 mg	200 mg
**pH**	6.99 ± 0.02	6.95 ± 0.01	6.96 ± 0.02	7.03 ± 0.07	7.09 ± 0.04 *	7.01 ± 0.04	6.98 ± 0.03
**NH_3_-N (mmol/g)**	5.32 ± 0.04	5.15 ± 0.05 *	5.15 ± 0.05 *	5.08 ± 0.04 **	5.06 ± 0.02 **	5.26 ± 0.07	5.15 ± 0.05 *
**Total VFA (mmol/g)**	122.42 ± 2.40	92.15 ± 2.64 **	80.52 ± 3.85 **	92.86 ± 10.7 **	49.54 ± 0.46 **	137.53 ± 0.03 ǂ	124.65 ± 2.99
**BCFA (%VFA)**	3.32 ± 0.13	3.56 ± 0.19	2.96 ± 0.18	3.70 ± 0.33	6.20 ± 0.26 **	3.47 ± 0.11	2.30 ± 0.17 **
**A/P**	3.08 ± 0.10	2.88 ± 0.06	2.95 ± 0.29	3.42 ± 0.27	4.26 ± 0.25 **	2.44 ± 0.30 ǂ	3.15 ± 0.17

**Table 4:** Effects of *F. sylvatica* Fs/1/1 extract and its fractions Fs/2/1 and Fs/3/2 at 50-mg and 200-mg dose levels on fermentation end products after 120 h of incubation. AcA = acetic acid; PrA = propionic acid; ButA = Butyric acid; ValA = valeric acid; iso-ButA = iso-butyric acid; iso-ValA = iso-valeric acid. * *p* < 0.01, ** *p* < 0.001, and ǂ *p* < 0.05. In the lower panel, the percentage increase or decrease of each volatile fatty acid was plotted for the different tested dose levels (◼ 50 mg and ◼ 200 mg) vs. FA% in the control diet.

% VFA	Control Diet	Fs/1/1		Fs/2/1		Fs/3/2	
		50 mg	200 mg	50 mg	200 mg	50 mg	200 mg
AcA	65.33 ± 0.53	65.89 ± 0.06	62.90 ± 2.47 ǂ	67.63 ± 1.35 ǂ	61.59 ± 1.23	68.62 ± 1.38	66.24 ± 0.47
PrA	21.18 ± 0.60	23.38 ± 0.69	21.39 ± 1.30	19.86 ± 1.18	14.47 ± 1.24 **	28.47 ± 2.99 **	21.04 ± 1.00
ButA	7.37 ± 0.15	8.36 ± 0.22	5.59 ± 0.45	7.84 ± 0.75	4.61 ± 2.58	10.08 ± 0.04	8.38 ± 0.41
ValA	1.90 ± 0.05	1.82 ± 0.22	1.45 ± 0.06 ǂ	1.20 ± 0.05 **	1.15 ± 0.05 **	1.76 ± 0.14	1.83 ± 0.09
iso-ButA	1.57 ± 0.07	1.21 ± 0.04	0.89 ± 0.11 *	1.16 ± 0.02 ǂ	1.22 ± 0.01	1.98 ± 0.42 ǂ	0.93 ± 0.03 *
iso-ValA	2.57 ± 0.02	2.07 ± 0.16 ǂ	1.50 ± 0.15 **	2.09 ± 0.16 ǂ	2.15 ± 0.32	2.62 ± 0.23 *	2.02 ± 0.10
	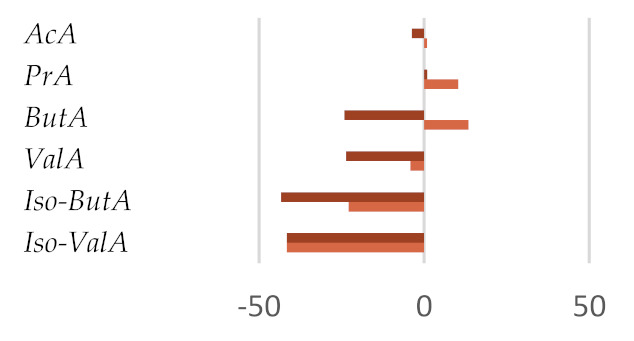			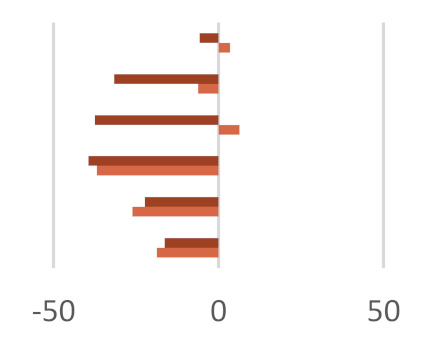		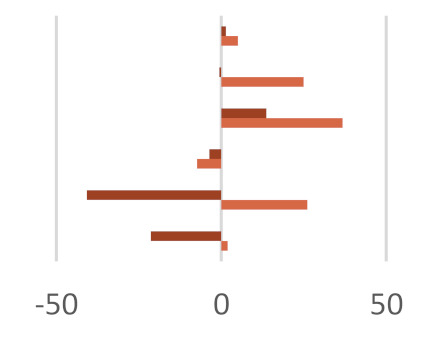	

## Data Availability

Data are within the manuscript and related Supplementary Materials.

## Supplementary Materials

The following supporting information can be downloaded at https://www.mdpi.com/article/10.3390/molecules27072217/s1, Figure S1: UV spectra of Fs/2/1 and Fs/3/2 beech leaf fractions, Figure S2: Heatmap of the correlation using Pearson’s coefficient correlation between antiradical (DPPH^●^ and ABTS^●+^) activities, reducing the activity (PFRAP), total phenol content (TPC), total flavonoid content (TFC), and total saponin content (TSC) with the fermentation parameters at the dose level of 50 mg, Figure S3: Heatmap of the correlation using Pearson’s coefficient correlation between antiradical (DPPH^●^ and ABTS^●+^) activities, reducing the activity (PFRAP), total phenol content (TPC), total flavonoid content (TFC), and total saponin content (TSC) with the fermentation parameters at the dose level of 200 mg.

## Sample Availability

Samples of the compounds are available from the authors.
