# Supplementary material for: UHPLC-ESI-QqTOF Analysis and In Vitro Rumen Fermentation for Exploiting Fagus sylvatica Leaf in Ruminant Diet
Source: Molecules. 2022 Mar 29;27(7):2217. doi: 10.3390/molecules27072217 (PMC9000816; doi:10.3390/molecules27072217)

UHPLC-ESI-Q*q*TOF analysis and *in vitro* rumen fermentation for exploiting *Fagus sylvatica* leaf in ruminant diet

Marialuisa Formato[1], Simona Piccolella[1], Christian Zidorn[2], Alessandro Vastolo[3], Serena Calabrò[3], Monica Isabella Cutrignelli[3], and Severina Pacifico[1,*]

Supplementary materials

Figure S1. UV spectra of Fs/2/1 and Fs/3/2 beech leaf fractions.

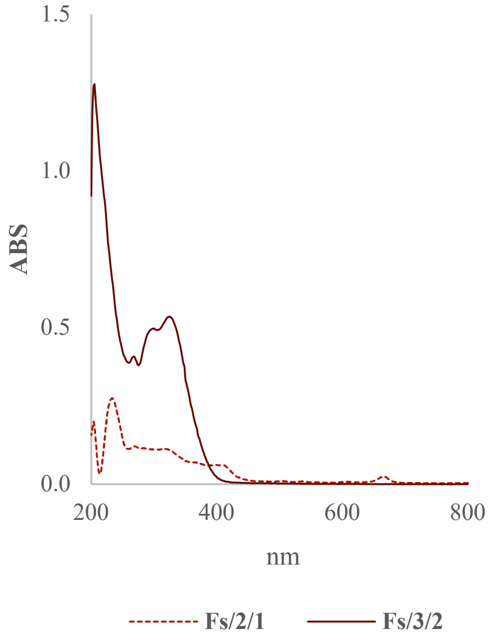

Figure S2. Heatmap of correlation, using Pearson's coefficient correlation, between antiradical (DPPH•, ABTS•+) activities, reducing activity (PFRAP), total phenol content (TPC), total flavonoid content (TFC) and total saponin content (TSC) with fermentation parameters at the dose level of 50 mg.

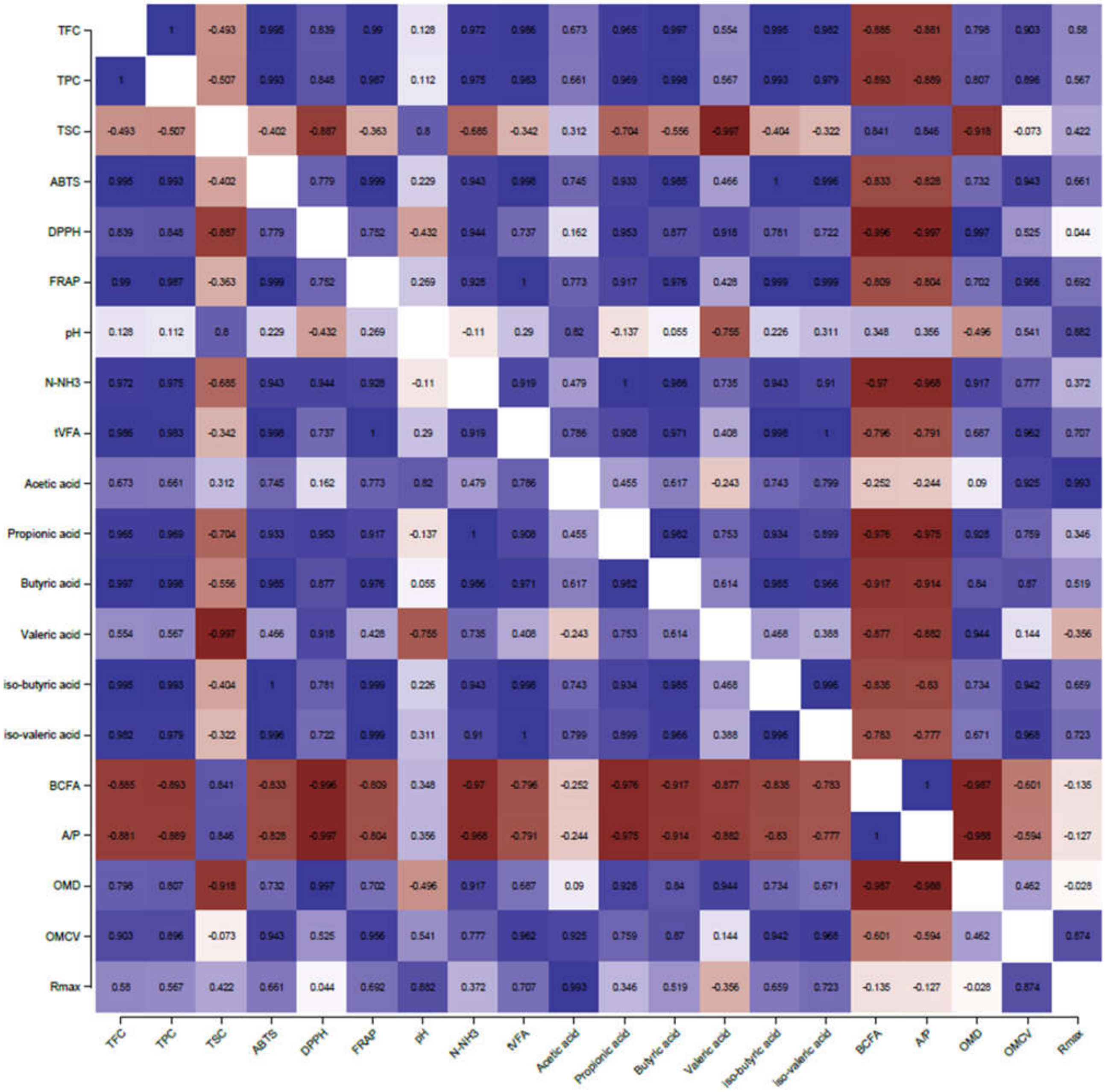

Figure S3. Heatmap of correlation, using Pearson's coefficient correlation, between antiradical (DPPH•, ABTS•+) activities, reducing activity (PFRAP), total phenol content (TPC), total flavonoid content (TFC) and total saponin content (TSC) with fermentation parameters at the dose level of 200 mg.

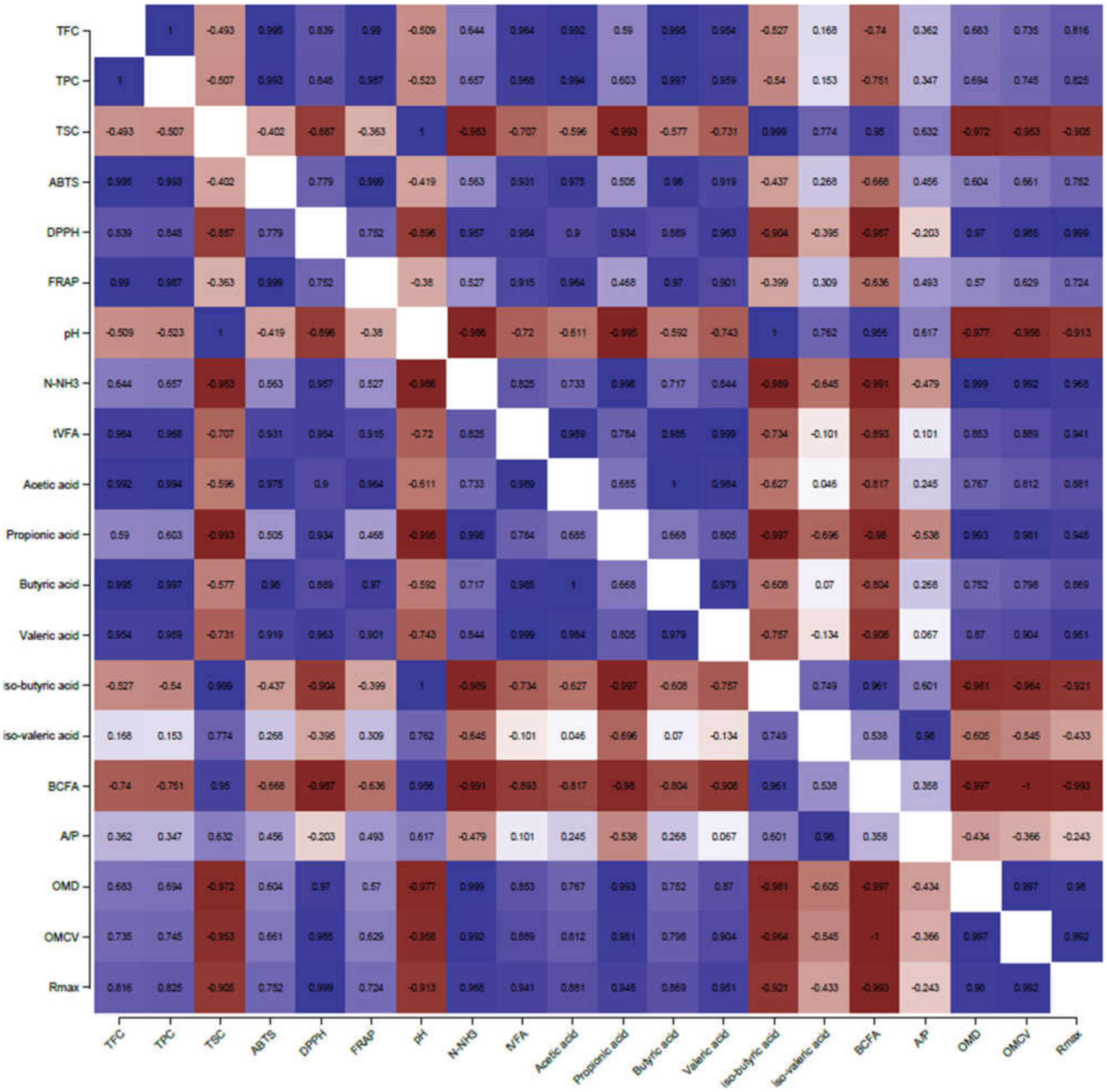

Supplement: Supplementary file 1 [file molecules-27-02217-s001.zip › molecules-1635605-supplementary.pdf]
